# *The Organon* and Materia Medica Club of the Bay Cities of California

**Published:** 1895-09

**Authors:** Eleanor F. Martin

**Affiliations:** Secretary pro tem.


### *THE ORGANON* AND MATERIA MEDICA CLUB OF THE BAY CITIES OF CALIFORNIA.

The regular semi-monthly meeting was held at the office of Dr. J. M. Selfridge, in Oakland, June 7th, 1895.

The members present were Drs. J. M. Selfridge, E. W. Bradley, A. McNeil, M. T. Wilson, S. E. Chapman, George H. Martin, George J. Auger, and C. M. Selfridge, and T. C. Coxhead visiting.

The meeting was called to order at 8.15 P. M. by the President, Dr. J. M. Selfridge.

The Secretary being absent, no minutes were read. The President then appointed Dr. McNeil to act as reader of the evening, who read from *The Organon*, Section 185.

### DISCUSSIONS.

Dr. Chapman—Do the old school teach that local affections are independent of a general condition?

Dr. McNeil—Certainly they do. These cuttings and suppressions of eruptions are harmful. Professor Hebra makes an illustration by saying that these eruptions are no more a part of the general condition than the dirt on your coat. Local treatment is certainly absurd and leads to harm. When we treat locally we suppress.

Dr. Bradley—Suppose the indicated remedy were used locally, would it then suppress?

Dr. McNeil—The indicated remedy applied locally would be apt to act more quickly than if given internally, and the external evidences of the disease will be removed, but the internal condition will remain, and hence the mischief is done, for now the most prominent symptoms to prescribe for are gone. I will cite a case. Two girls came to my office; one about twelve years of age, who was puffing so much that I thought she had been running hard. She said not, but that she had asthma. To my question, "Did you ever have an eruption on the head?" she answered: "No; it was on my hands." After more questioning I concluded that the asthma was due to the suppression of this eruption. The suppression of eruption is a frequent cause of asthma and other diseases. In nasal catarrh, nasal douches or local applications are often supposed to cure, but soon after throat and lung affections present themselves. Catarrh does not kill; suppress it, and it goes inward and strikes some vital spot.

Dr. Chapman—Not long since I had a splendid action of Sulphur. A lady with cancer of the right breast (scirrhus); she had also a hard lump in the right side of the neck, like goitre. Her symptoms were clearly and distinctly Sulphur. I gave $\text{Sulphur}^{\text{cm}}$, and in a few days she presented a perfect picture of measles, and the other symptoms soon began to abate. This was three months ago, and now there is every appearance of recovery. Could the suppression of measles in this woman as a child bring about the condition of the neck and breast?

Dr. McNeil—Yes.

Dr. Wilson—Would the remedy that brought out the measles cure the disease without suppressing it, if given in childhood during the attack of measles?

Dr. McNeil—Yes.

Dr. Chapman—I am astonished that the old school still keep up this local treatment. I thought that they had outlived this idea of treatment. Cancer has always been considered a local disease by them, and their treatment for it has always been the knife, first, last, and all the time.

Sections 186–191 were then read.

### Discussions.

Dr. Martin—The allopaths to-day do treat these skin diseases locally, and while they have found out that the eruption is simply the external manifestation of an internal nerve irritation which prevents the skin from being properly nourished; and while this is a step forward in their pathology, yet they do not take advantage of that knowledge in their treatment, but simply use the local measures, almost to the exclusion of internal means, and the reason they have thought of the internal cause of this external disease is because the homœopaths have had to think the matter out themselves, and the old-school physicians have profited by it. Even in chorea they simply give remedies to tone up the nervous system, and thus suppress the external manifestations of the disease.

Dr. Chapman—What is their success in chorea?

Dr. Martin—They suppress the disease for a time, and seem to cure quicker than we do; but it returns again inside of six months, and in some cases returns many times. Dr. Dana, of New York, says that the disease frequently returns and occasionally other diseases come on in its place.

Dr. Bradley—A case of chorea was under my care which did not improve. It went to an old-school physician, who gave Chloral-hydrate and Bromide of Potassium, which cured. The patient is now in State prison.

Dr. Chapman—I had a case of a boy fourteen years of age, who had been in allopathic hands for two years. He did tolerably well for a time, then became very bad, and came to me. I tried several remedies, and at last gave Migale$^{30}$, which did remarkably well.

Dr. J. M. Selfridge—Here is a case: A boy ten years of age with chorea which had been preceded by an eruption on the skin which had been suppressed by the use of Zinc ointment locally applied. The skin disease looked very much like that of lepra. A few months after it disappeared chorea commenced. I told the parents that I would like very much to bring out the skin disease again. I gave Bryonia to throw it out again, which it did, with the result that I was not wanted any more. The chorea was due to the suppression of the rash in this case. The allopaths do treat skin diseases as if they were local.

Dr. Martin—They have all kinds of applications. I have seen a great many cases supposed to be cured by these men, and yet these nerve symptoms come on later. Eczema capitis is the most dangerous when local treatment is used. The poison must react some way. There are a great many chronic nervous diseases due to these conditions. If the old-school men would follow these cases up they would see how bad the results are. I have used Migale in cases of chorea with splendid results.

Dr. Wilson—I had a case of eczema capitis in a little girl. She had been treated by allopaths in the Samoan Islands, who suppressed the eruption, after which she became very sick, and remained so until the eruption appeared again. She came to me, and I gave her Sulphur$^{30}$, which brought it all out again until her head was one whole scab. I had to put a net over the head to keep the flies off. As soon as the eruption commenced to come out she began to improve, and kept on getting better, until finally she was entirely cured by the Sulphur$^{30}$.

Dr. Auger—Speaking of allopaths treating skin diseases locally and not internally, I will say that when I was an allopath I treated locally but also constitutionally, as I thought the local disease was due to some constitutional taint, as heredity or the like. I used Arsenic, Iodide of Potash, or some remedy to get an alterative effect, and in this way counteract the constitutional taint. I found that sometimes these conditions were kept up by troubles of the digestive organs, torpid liver, bowels, etc., hence I treated these troubles, too. As far as my treatment in those days is concerned, I did not believe it local trouble entirely, and therefore treated both externally and internally.

Dr. Martin—That is why Dr. Auger is a homœopath now; as his statements show that he did more thinking than is usually done by old-school practitioners regarding these conditions.

Dr. J. M. Selfridge—Is that the general trend of the allopaths now?

Dr. Auger—I think not.

Dr. Bradley—I am well acquainted with Dr. J. Austin Miller, of Oakland, and he claims that allopaths do consider these conditions general as well as local, and that a large proportion of these cases are given Sulphur internally with an ointment externally.

Dr. Chapman—I had prairie itch when a boy. I was given Sulphur both inside and out by allopaths until I declared that

I would not have any more of it. I then treated myself by putting my hands into a bowl of hot water and allowing them to stay there for some time, as it seemed to relieve the terrible itching more than anything else; and I finally cured myself with this treatment.

Dr. McNeil—Professor Hebra and the men at the University of Vienna treat them all locally. There is a tendency, however, of allopaths coming our way. The term "nervous" is not a clear expression as a cause of skin diseases. The French dermatologists have come to our views as to the psoric origin of skin diseases. This is the sycotic theory.

Dr. J. M. Selfridge—They call it a dyscrasia.

Dr. Chapman—Do they not now teach that the surgical treatment of fistula in ano will result in phthisis?

Dr. McNeil—Yes; this statement may be found in Osler. He says that if consumption be present the operation hastens death.

Dr. Martin—Years ago they used to say that the stoppage of chronic ear discharge would cause consumption.

Dr. C. M. Selfridge—Also that the curing of chronic ulcer of the leg would produce the same result.

Dr. McNeil—I had a case of a woman who had an indolent ulcer on one of her legs for thirty years, alternating with colic. There was not much pain in the ulcer, but profuse discharge. It was often healed by external applications, but would be invariably followed soon after by colic so severe that often for five days and nights she would have no sleep. I cured her with Calcarea-carbonica$^{200}$. This remedy covered both the ulcer and the colic. Six years after she had a slight return. I sent her more of the medicine, and she has since remained cured. How could she have had two diseases without any apparent connection? She had *one* disease which manifested itself in *two* ways.

Dr. Martin—It was the *skin phase* of the disease. I have a man under my care suffering from rheumatism in the ankles, knees, and wrists. He had large, impetiginous, ulcerated spots on the wrists. Local applications had been used which cured the ulceration, but made the rheumatism worse. Sulphur$^{200}$ was indicated, which I gave, and the patient is now doing well.

Dr. C. M. Selfridge—I had a case of a man with occasional attacks of colic, associated with a 5 A. M. diarrhœa. He had an accident to one of his legs, knocking it against a timber. A sore appeared on the shin, and then he had no more colic. I gave Sulphur for a year. The patient said that his father had had a similar sore.

Dr. Chapman—The old-school men formerly used a seton for the relief of various conditions, and claimed they had fine results.

Dr. McNeil—When they gave up the seton, then they made a mistake.

Dr. J. M. Selfridge—In regard to the continuity of disease: I had a case of eczema of the head and face in an infant. I thought it a Rhus case. The mother had applied tar ointment, with the result that the rash soon dried up, the little one became drowsy, and cerebral symptoms developed. The mother, being an intelligent woman, inferred that the drying up of the rash was the cause of the cerebral symptoms. She took off the application, and the patient got better; then came to me, and I gave it the indicated remedy to cure.

Sections 192–195 of *The Organon* were then read.

### Discussions.

Dr. McNeil—A point comes in here: When we are called to a case of acute disease. If we are called early and give the indicated remedy, it will usually cure. If we are not called early, we cannot cure the acute trouble, but we must cure the psora. In scarlatina, for instance, if called early and give the indicated remedy, that will be the end of the treatment. Sac-lac. will do the rest of the work for weeks. If we are called later in the disease, the internal psora may have been aroused into action, and we will then have the sequelæ to look out for. The sequelæ are simply the effects of the aroused psora.

Sections 196, 197 of *The Organon* were then read.

### Discussions.

Dr. Bradley—In regard to the sequelæ of scarlatina—post-scarlatinal dropsy, for instance. Is it psora or the poison of scarlatina passing through the kidneys which causes the dropsy?

Dr. McNeil—You now get down to a material cause of the disease, and it will not bear investigation. The indicated remedy given promptly will cure. The latent psora does not need to be cured, but if aroused it attacks the kidneys, and hence the psora must now be cured.

Dr. Chapman—In any case of scarlatina before desquamation is complete, if cold is taken does it not cause nephritis?

Dr. McNeil—The mortality in eruptive fevers is not any greater in the slums than among the higher classes. This is also so in Vienna and other parts of Europe, where the peasantry is much worse off than our poor here. *It is the psora*, not the cold, which causes the dropsy.

Dr. J. M. Selfridge—From my observations, the sequelæ do not come for two weeks after the scarlatinal rash disappears. Exposure would predispose the nerves to irritation, as in burns nephritis may be caused by the removal of the cuticle.

Dr. Martin—That is just exactly the case. I have never had any of the sequelæ of scarlatina, but I keep my patient confined to the room for six weeks, until desquamation has entirely ceased. The skin is sensitive, and exposure to cold would produce these effects as in eczema. Ulceration of the bowels often follows burns, as the internal organs are extremely sensitive to disease, for they feel the effects of the congestion. Another disease, diphtheria, produces a neuritis due to the effects of the diphtheritic poison upon the nerves, in the same manner that Mercury, Lead, and Arsenic produce inflammation of the nerves. Diphtheritic paralysis is common in the old school, but not in ours.

Dr. McNeil—I admire the logic of the gentleman, but logic is often overturned by effects. In the Franco-Prussian war large numbers of typhoid-fever cases were treated in the open air in the streets. It rained frequently, and yet the results were the best of any ever known.

Dr. Martin—There are just as many cases of cold on Nob Hill as there are on Tar Flat, and the illustration given by Dr. McNeil does not cover the case at all.

Dr. Chapman—I do not see the application of Dr. McNeil's remarks.

Dr. McNeil—It is the same in any of these diseases; they say if there is any exposure to cold they have it. Nephritis does not arise from cold; *it comes from psora.* If you give the indicated remedy in time they will not have the sequelæ.

Dr. J. M. Selfridge—Speaking of cold, here is a case: A sea-captain's daughter, sixteen years of age, was attacked with measles. She was sitting in the doorway of her state-room with an open window behind her, which caused a slight breeze to blow upon her. The measles receded, convulsions came on and she died. It was like driving in an eczema, and it went to the brain.

Dr. Chapman—Could that have occurred if the patient had no psora?

Dr. McNeil—No.

Dr. Bradley—If a person is not sick, can you tell whether they have psora or not?

Dr. McNeil—If I examine for it I can usually find it. We nearly all have it more or less. Jim Corbett has it.

Dr. J. M. Selfridge—A great many have it, but not all. If we have not psora we have something else.

Dr. McNeil—If you commence now and take a careful report of your cases, questioning carefully regarding the personal and family history, you will find that there is psora in nearly every case.

Dr. J. M. Selfridge—I cannot think we all have it.

Dr. Martin then read Section 80, stating what psora is.

Dr. Bradley—Do all true homœopaths believe this psoric theory?

Dr. McNeil—Yes; and if not they are not true homœopaths. They must accept the psoric theory as a fundamental truth.

Dr. Martin—The whole trouble is that the meaning of the word is misunderstood. *It is the fundamental cause of all disease.* Some call it germs, bacilli, etc., but these are simply changes of name.

Dr. J. M. Selfridge—It is not the fundamental cause of every disease. Hahnemann gave other causes.

Dr. Martin—They are all the same.

Drs. J. M. Selfridge and McNeil—No; they are not.

Dr. Martin read again Section 80—*i. e.*, what psora is.

Dr. McNeil—They are separate and distinct.

Dr. Chapman—If gonorrhœa was suppressed, and there was no psora, would we have sycosis?

Dr. McNeil—Yes.

Dr. Martin read Section 80 again.

Dr. McNeil—I never understood it so.

Dr. Auger—Then one cannot have gonorrhœa or syphilis unless there is psora?

Dr. McNeil—I will not accept this until I go over it more carefully in the original German. I think there is a mistake here. How does this agree with the fact that Hahnemann has antipsoric, anti-syphilitic, and anti-sycotic remedies?

Dr. Martin—He simply classifies his remedies. If there is no particular term applied to disease, then no particular term will be required for the remedy. If not, it must be the antipsoric.

Dr. Chapman—Any remedy capable of curing a chronic disease is antipsoric.

Dr. McNeil—No; we may have a patient needing antipsoric, anti-syphilitic, or anti-sycotic remedies. Hahnemann tells us if necessary to give all three together; for he meant them to be distinct and separate.

Dr. J. M. Selfridge—Hahnemann recognized three distinct miasms. Psora may be mixed with the others, but still they are distinct.

Drs. Martin, Chapman, and Wilson—Psora is the base of all disease.

Section 198 was then read.

### Discussions.

Dr. J. M. Selfridge—Hahnemann's idea is that the indicated remedy should not be used externally, as it may suppress.

Dr. Chapman—Some teach that the local manifestations are not the principal symptoms to get. Hahnemann speaks of it as such.

Dr. McNeil—In looking up a case of ulcers in Bœnninghausen you look for the modalities and concomitants. It takes all to give the totality of symptoms. A man with ulcer thinks that is all of his trouble, and so it is with many. Dr. Dunham in a case of ovarian disease, said the symptoms seemed to indicate Cyclamen. It was given and cured. Then it was discovered that Cyclamen had never been proven on women.

Dr. Chapman—Section 153 tells us that prominent symptoms are the ones to be considered. In my experience sometimes it is the subjective, and at other times the local ones that give me the key for a remedy. Often there are no subjective symptoms, and then we have to judge by the local.

Dr. McNeil—If all the features of the case were present we could tell better what to give.

Dr. J. M. Selfridge—Even if we had the true *simillimum* it would act by suppressing the disease, without curing it if used externally.

Dr. Chapman—It acts more rapidly externally than internally, and hence takes away the prominent symptoms upon which we would prescribe.

Dr. McNeil—We must prescribe for all of the symptoms.

Section 199 was then read.

### Discussions.

Dr. McNeil—Bœnninghausen was the best explainer of Hahnemann, yet if we look for a remedy in his book for ulcers, for instance, we find under "ulcers," round, flat, and all kinds of ulcers, and we may cure a case by selecting a remedy from this group; and yet if we turn to "ameliorations" and "aggravations" we are liable to find other remedies that would seem to answer our purpose perhaps better.

Upon motion of Dr. Wilson, seconded by Dr. Chapman, the meeting then adjourned to meet again the third Friday in June at the office of Dr. McNeil in San Francisco, when the reading of *The Organon* would be continued, commencing at Section 201.

ELEANOR F. MARTIN, M. D.,
*Secretary pro tem.*

